# An Unexpected Complication After Extracorporeal Shock Wave Lithotripsy: Emphysematous Pyelitis

**DOI:** 10.7759/cureus.8307

**Published:** 2020-05-27

**Authors:** Kamil Gokhan Seker, Yusuf Arikan, Yurdagul Cetin Seker, Deniz Noyan Ozlu, Ismail Evren

**Affiliations:** 1 Urology, Bakirkoy Dr. Sadi Konuk Training and Research Hospital, Istanbul, TUR; 2 Emergency Medicine, Muş State Hospital, Muş, TUR; 3 Urology, Bakirkoy Dr. Sadi Konuk Training and Research Hospital, İstanbul, TUR

**Keywords:** emphysematous pyelitis, extracorporeal shock wave lithotripsy, eswl, urinary tract infection

## Abstract

Emphysematous urinary tract infections (UTI) are life-threatening conditions caused by gas-forming organisms. Emphysematous pyelitis (EP) is a rare, acute bacterial UTI characterized by gas formation only in the renal collecting system. Extracorporeal shock wave lithotripsy (ESWL) treatment was performed for 10-mm sized stone in the left renal pelvis in an 81-year-old female patient with no known comorbidities other than hypertension. In the 10th hour following ESWL treatment, the patient referred to the emergency department with fever and left flank pain. Gas was noticed in the left renal collecting system in non-contrast computed tomography (NCCT). A wide spectrum antibiotic was given to the patient due to EP diagnosis and a nephrostomy catheter was placed in the left renal pelvis. EP should be considered in the patient with fever and flank pain after ESWL and NCCT should be performed for further examination. Quick diagnosis, examination and treatment of these patients in the emergency department are important.

## Introduction

Extracorporeal shock wave lithotripsy (ESWL), commonly used for proximal ureteral and renal stones, is an effective and safe treatment. However, ESWL treatment has some complications. They occur mostly in the gastrointestinal, cardiovascular and urogenital systems and are usually related to symptoms due to residual stone fragments. Infectious complications have a wide range, including asymptomatic bacteriuria, urinary tract infection (UTI) or sepsis [1].

Infections characterized by gas in the urinary system are emphysematous cystitis (EC), emphysematous ureteritis (EU), emphysematous pyelitis (EP) and pyelonephritis (EPN), and all these infection types represent aggressive and potentially life-threatening conditions which require medical and sometimes surgical treatment. It often progresses to sepsis in the absence of early medical intervention. EP is an acute UTI characterized by gas only in the collecting system where there is no gas accumulation in the renal parenchyma and extrarenal area. EP cases generally have a quick recovery after the early intervention and present a good prognosis [2].

In literature, after ESWL treatment early period EPN and late period ES, EU and EP cases have been reported [3,4]. As far as we know, this is the first case presentation reporting early period isolated EP after ESWL treatment.

## Case presentation

**Figure 1:**
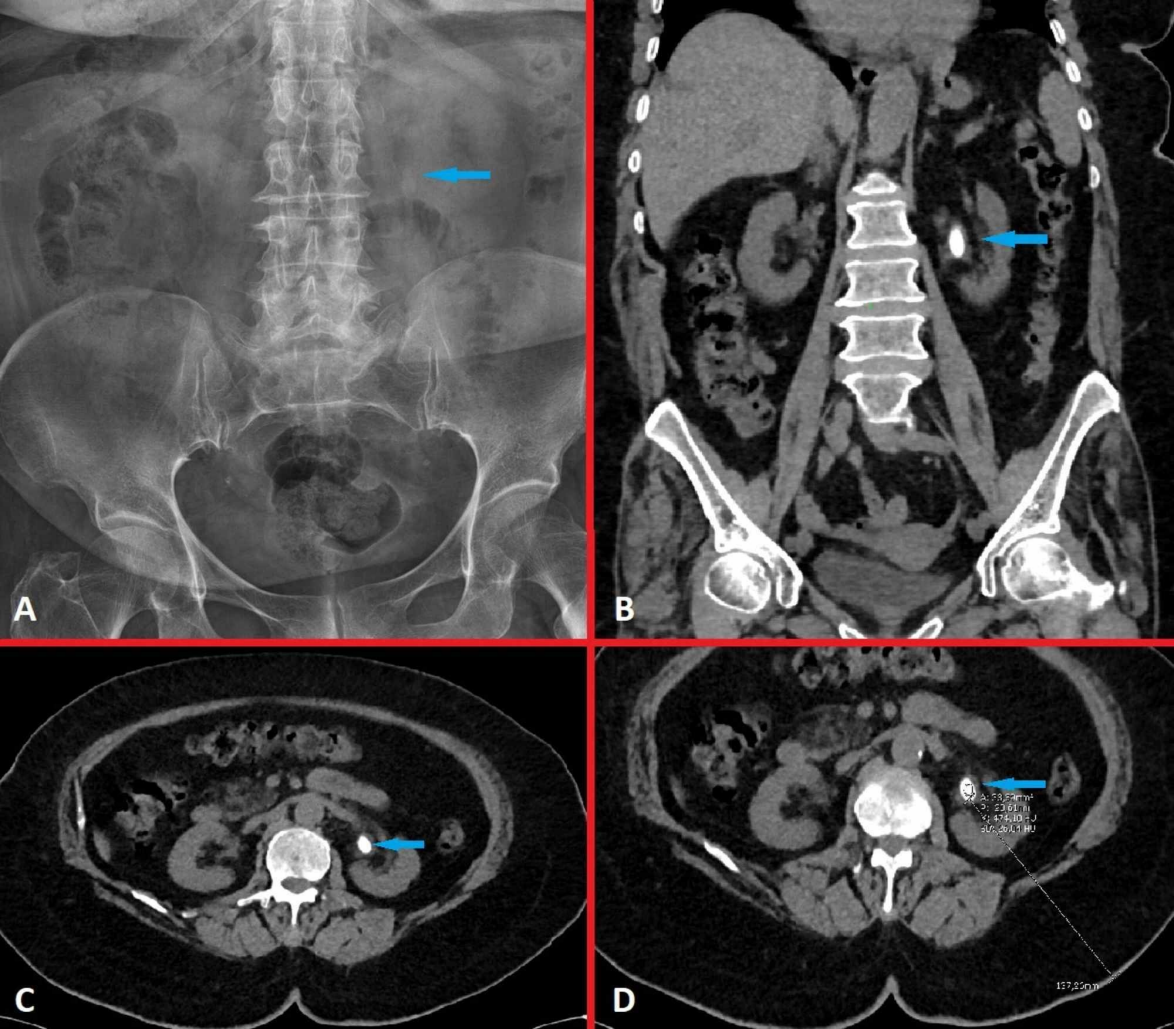
Radiological imaging before ESWL treatment A 10-mm sized semi-opaque stone in the left renal pelvis on KUB X-ray (A), in coronal (B) and in transverse cross-sections (C) on NCCT. Stone density (440 Hounsfield units) and skin to stone distance (13 cm) were shown in transverse cross-section on NCCT (D). ESWL: extracorporeal shock wave lithotripsy, KUB: kidney-ureter-bladder, NCCT: non-contrast computed tomography.

An 81-year-old female patient was admitted to the urology outpatient clinic with complaints of left flank pain. The patient had no additional comorbidities other than hypertension. Left costovertebral angle tenderness (CVAT) was detected in the physical examination. Serum creatinine, infectious parameters and urine analysis values were within the normal range. The urinary culture was sterile. A 10-mm sized stone was observed in the left renal pelvis on ultrasonography (USG). The stone was confirmed by a kidney-ureter-bladder (KUB) X-ray, intravenous pyelography (IVP) and non-contrast computed tomography (NCCT) (Figure 1).

**Figure 2:**
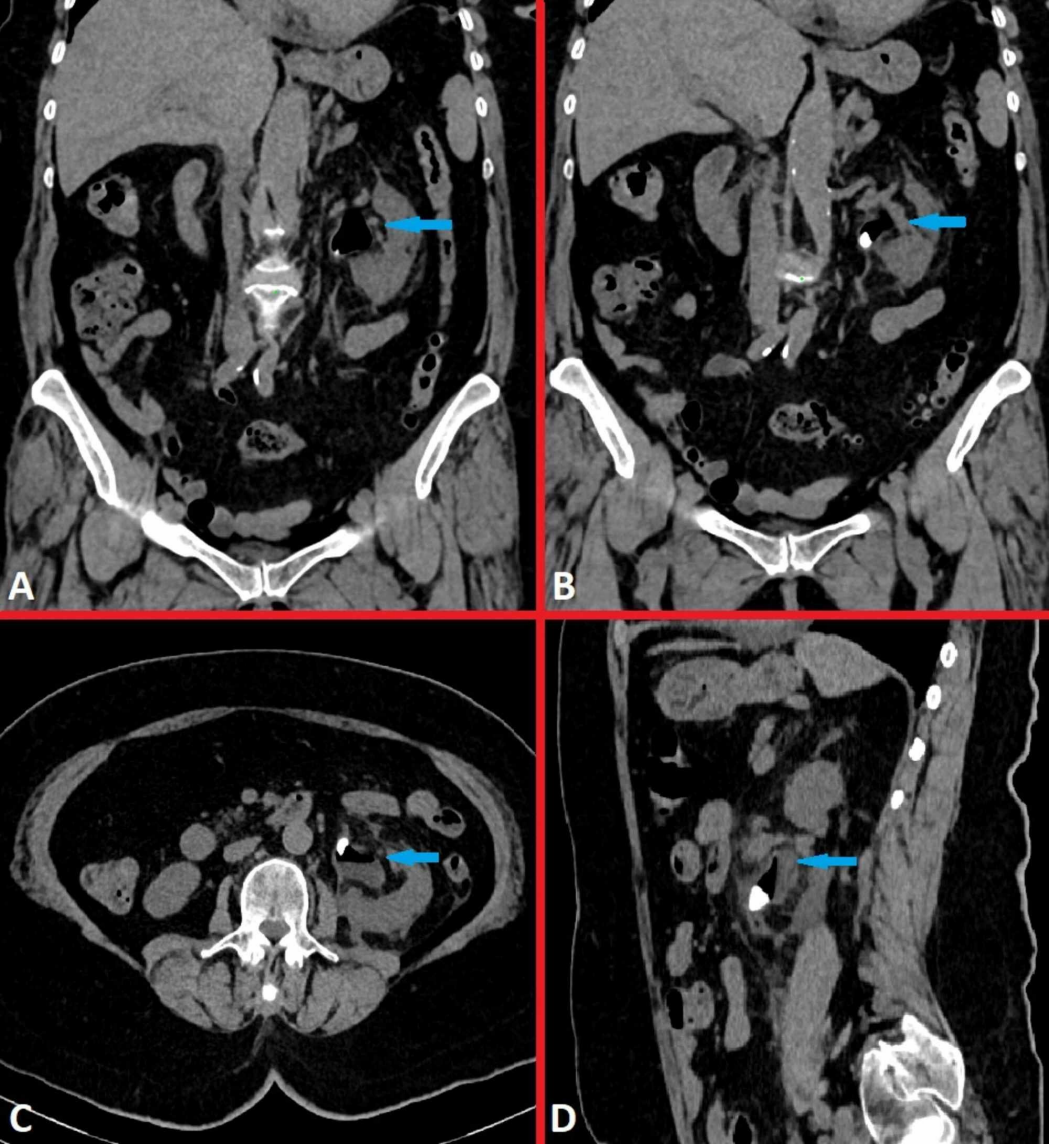
Radiological imaging during admission to the emergency room A 10-mm sized stone and gas image in the left renal pelvis, in coronal (A,B), in transverse (C) and in sagittal cross-sections on NCCT ((D). NCCT: non-contrast computed tomography.

ESWL treatment was planned for the patient. No complications were detected during the procedure in the patient who underwent the first session of ESWL treatment. The patient was admitted to the emergency department at the 10th hour after ESWL with complaints of fever and left flank pain. When vital signs were examined, body temperature was 38.7 °C, blood pressure was 110/70 mgHg, respiratory rate was 22 breaths/min and the heart rate was 90 beats/min. A significant CVAT was detected on the left side of the patient in the physical examination. Based on laboratory findings, creatinine level was 1.82 mg/dL (normal: 0.6-1.1 mg/dL), C-reactive protein (CRP) was 33 mg/L (normal: 0-5 mg/L) and white blood cell (WBC) count was 15.3 × 10e3/uL (normal: 4.5-10 × 10e3/uL). Pyuria was detected in the urinary analysis. Grade 1 hydronephrosis was detected on the left in USG. In NCCT, a stone in the left kidney renal pelvis and a significant gas image in the pelvicalyceal system were observed (Figure 2).

The diagnosis of EP was considered in the patient. The patient was hospitalized with empirical broad-spectrum antibiotherapy (third-generation cephalosporin-ceftriaxone 2 gr/day). As the fever persisted during follow-up, a nephrostomy catheter was placed in the left kidney by the interventional radiology department. In the urine culture taken from the nephrostomy catheter, multidrug-resistant Escherichia coli was detected; thus, ertapenem treatment was applied by the department of infectious diseases. After nephrostomy placement, infectious parameters and the clinic of the patient regressed, and the patient was followed up under ertapenem 1 gr/day treatment for 14 days without any complications. A month later, flexible ureterorenoscopy (URS) was performed for left renal pelvis stone, the left nephrostomy catheter was removed and a ureteral stent was placed. Complete stone-free condition was provided.

## Discussion

Emphysematous UTI after ESWL is a quite rare life-threatening condition. Only a few cases have been reported in the literature. This case presentation is the first presenting an early period isolated EP after ESWL.

Similar to EPN, EP is mostly related to uncontrolled diabetes mellitus and urinary system obstructions.* *EP is a milder form with a better prognosis, where the gas is limited to the pelvicalyceal system and is often associated with obstructive uropathy due to stone, stenosis and neoplasm [5].

In a study investigating the risk factors for the development of pyelonephritis after ESWL, it was thought that ureteral obstruction caused by the stone fragments and bacteria in the stone were important [6]. Also, the microhemorrhages in small renal arteries during ESWL may cause the release of inflammatory response cells and from these microhemorrhages, bacteria in the stone can enter the bloodstream [7]. EP clinic occurring after ESWL treatment in our case without any additional comorbidities makes us consider that acute obstruction following endourological interventions may constitute a risk factor for EP. It is thought that the bacteria released after lithotripsy play a role in the patient with a sterile preoperative urine culture.

Approximately 69% of EP cases are due to Escherichia coli infection. Other common factors are Klebsiella pneumonia and Aerobacter [8]. In our case, although urine culture taken before ESWL was sterile, multidrug-resistant Escherichia coli was detected in the sample taken from the nephrostomy catheter.

EP is mostly seen in women (F/M ratio 5:1). Fever, hematuria, dysuria, vomiting and side pain were observed during the admittance of the patients. Clinical presentation is milder compared to EPN patients. Although the clinic of EP is more stable, there may also be severe sepsis presentation [9]. Our case was a female patient who had a fever, shivering and CVAT, but she did not have a clinical sepsis presentation.

The rate of detection of EP or EPN by KUB X-ray is low because it is difficult to separate them from intestinal gas [8]. Although it is misleading in USG, it may be observed in a careful analysis that the posterior acoustic shadowing formed by gas is dirtier compared to the shadowing caused by stone [2]. NCCT is the most sensitive and specific imaging method for the diagnosis of emphysematous UTI. It is reliable for demonstrating the presence and location of the gas in the collecting system, renal parenchyma and perinephric spaces. It also helps in the separation of EPN and EP [8]. In our case, although no gas was detected in the USG taken in the emergency room, in NCCT, gas was seen only in the collecting system, and no gas was observed in the renal parenchyma and perinephric areas.

The first treatment of emphysematous UTI, as in EPN, involves fluid and electrolyte resuscitation, broad-spectrum antibiotic therapy targeting gram-negative bacteria and control of diabetes if present [8]. After providing hemodynamic stability, renal function test, hematological and basic laboratory parameters including blood glucose level should be evaluated. Urine, blood and sensitivity tests should be performed for microbial culture. A quick response can be achieved by conservative treatment. However, drainage is important in obstructed urinary systems [10].

Emphysematous UTI can be progressive and fatal, requiring aggressive intervention. The mortality rate of EP is 20%, which is lower than that of EPN [8].

In literature, Wong and Pace reported EP in addition to EU and ES a few weeks after ESWL in a 36-year-old male patient with gout history, non-obstructive megaureter and congenital solitary right kidney. They achieved successful results with antibiotherapy and a nephrostomy catheter [4]. Kim et al. performed simple nephrectomy in a high-risk patient who developed EPN after ESWL [3]. Our patient, who received third-generation cephalosporin and supportive treatment in the early period in the emergency room, achieved a successful result with the insertion of a nephrostomy catheter and an ertapenem treatment suitable for his urine culture, and the patient's clinic was completely recovered.

## Conclusions

Patients presenting with fever and flank pain after ESWL should be evaluated for the appearance of gas with an NCCT scan; although rare, EP should be kept in mind. Quick diagnosis, examination and treatment of these patients in the emergency department are important. Successful results can be achieved through medical treatment and percutaneous nephrostomy catheter.
